# NLRP6 Inflammasome Ameliorates Brain Injury after Intracerebral Hemorrhage

**DOI:** 10.3389/fncel.2017.00206

**Published:** 2017-07-14

**Authors:** Peng-Fei Wang, Zhen-Guang Li, Yong Zhang, Xiao-Hua Ju, Xin-Wu Liu, Ai-Ming Zhou, Jing Chen

**Affiliations:** ^1^Department of Neurology, Weihai Municipal Hospital Weihai, China; ^2^Department of Neurology, The Third Hospital of PLA Baoji, China

**Keywords:** intracerebral hemorrhage, inflammasome, neuroinflammation, NLRP6, brain injury

## Abstract

NLRP6 inflammasome, one of the important intracellular innate immune sensors, has been shown to regulate immune responses. However, its roles in the intracerebral hemorrhage (ICH) are completely not clear. In the present study, we investigated the expression profile and biological roles of NLRP6 inflammasome in perihematomal brain tissues of mice subjected to ICH. In this study, we investigated the expression profile of NLRP6 inflammasome in the perihematomal brain tissues and explored the biological role of NLRP6 inflammasome upon acute brain injury in mice subjected to ICH. Increased expression of NLRP6 inflammasome was found in perihematomal brain tissues ranging from 6 h to 3 days, with a peak level at 1 day after ICH. Immunohistochemistry staining also showed that NLRP6 inflammasome was significantly increased in the perihematomal brain tissues at 1 day after ICH. Moreover, immunofluorescence staining showed that NLRP6 inflammasome was mainly colocalized in glial fibrillary acidic protein (GFAP)-positive astrocytes, while with little colocalized expression in NeuN-positive neurons and without expression in CD11b-positive microglia and CD31-positive endothelial cell in the perihematomal brain tissue of mice after ICH. Furthermore, NLRP6^−/−^ ICH mice exhibited significantly higher brain water contents (BMCs), proinflammatory cytokines, NF-κB activity and neurological deficit scores when compared with the wild type (WT) ICH mice. In addition, we found that Toll-like receptor 4 (TLR4)^−/−^ mice, as well as the TAK242 treated mice, had markedly lower expression of NLRP6 inflammasome expression in the perihematomal brain tissue at 1 day after ICH. Our data suggest that the upregulated NLRP6 inflammasome in perihematomal brain tissues attenuates ICH-induced brain injury.

## Introduction

Intracerebral hemorrhage (ICH) is characterized by high mortality and morbidity and accounts for 23.8% of all strokes in China (Zhou et al., 2014; Wang et al., 2017). However, there is still no effective treatment for ICH (Qureshi et al., 2001; Zhou et al., 2014).

Converging evidence suggests that sterile inflammatory injury contributes to ICH-induced brain damage (Zhou et al., 2014; Xiong et al., 2016b). The enhanced production of proinflammatory cytokines released by activated glial cells and infiltrated leukocytes after ICH was the major reasons for inflammatory injury (Mracsko and Veltkamp, 2014; Wei et al., 2014; Zhong et al., 2016). Among these proinflammatory cytokines, interleukin (IL)-1β is considered as a pivotal therapeutic target in ICH (Masada et al., 2001; Lok et al., 2012). Recently, increasing evidence has suggested that the maturation of proinflammatory cytokines such as IL-1β to engage innate immune defenses was triggered by the inflammasomes (Schroder and Tschopp, 2010; Lamkanfi and Dixit, 2014). Inflammasome, as one of the intracellular innate immune sensors, has been shown involved in the pathogenesis of sterile inflammatory response following central nervous system (CNS) disorders, such as traumatic brain injury (Tomura et al., 2012), ischemic stroke (Abulafia et al., 2009) and ICH (Ma et al., 2014).

Members of the intracellular nucleotide-binding and oligomerization domain (NOD)-like receptor (NLR) family contribute to immune responses through activation of inflammasome signaling (Kanneganti et al., 2007). The NLR family member NLRP6 inflammasome was recently shown to negatively regulate innate immunity (Anand et al., 2012) and the activity of NLRP6 was critical for protection against inflammation-related colon tumorigenesis (Chen et al., 2011). Additionally, NLRP6 inflammasome was also reported contributing to recovery from peripheral nerve injury by dampening inflammatory responses (Ydens et al., 2015). These results strongly suggested that NLRP6 inflammasome may play important roles in regulating inflammatory injury to defense against the damages, but little is known about the function of NLRP6 inflammasome in ICH.

In addition, NLRP6 inflammasome has been shown to be enhanced by lipopolysaccharide (LPS) stimulation (Lee et al., 2015), which may be related to the activation of the Toll-like receptor 4 (TLR4) signaling by LPS. Given the TLR4 signaling has been demonstrated activated after ICH (Fang et al., 2013), therefore we hypothesized that ICH activated TLR4 signaling may influence the NLRP6 inflammasome expression and play an important role in the brain injury caused by ICH.

In the present study, we investigated the expression profiles and biological role of NLRP6 inflammasome in ICH-induced brain injury. We found that NLRP6 inflammasome expression was significantly enhanced and mainly colocalized in the glial fibrillary acidic protein (GFAP)-positive astrocytes after ICH, and increased NLRP6 inflammasome that may be regulated by TLR4 signaling attenuates ICH-induced brain injury.

## Materials and Methods

### Animals

All procedures involving animals were approved by the Animal Studies Committee of Third Hospital of PLA and Weihai Municipal Hospital and complied with the Guide for the Care and Use of Laboratory Animals (Guide; NRC 2011). In this study, wild type (WT) mice were purchased from the Animal Center of the Third Military University (Chongqing, China), TLR4^−/−^ were purchased from the Model Animal Research Center of Nanjing University (Nanjing, Jiangsu, China). According to a previously reported method (Chen et al., 2011; Anand et al., 2012), NLRP6^−/−^ mice were generated by the replacement of exons 1 and 2 of the Nlrp6 gene (N-terminal domain) with the internal ribosome entry site–β-gal–neomycin resistance cassette using a targeting vector (with the help of Beijing Biocytogen Co., Ltd). Eight to ten-week-old or 22 ± 2 g mice were used in study. All mice had a C57BL/6 background, and only male was used. The mice were provided food and water *ad libitum*, and were housed under a 12 h light and 12 h dark cycle.

### ICH Models

The detailed procedures used to construct the ICH model were established in our previous studies (Wang et al., 2013; Xiong et al., 2016a). Mice were briefly anesthetized intraperitoneally with 4% phenobarbital sodium and were immobilized on the stereotaxic apparatus (RWD Life Science Co, Shenzhen, China). All experimental mice received a total of 20 μL autologous blood injected into the caudate nucleus (bregma 0: 0.8 mm anterior, 2 mm left lateral and 3.5 mm deep) successively. The needle of microsyringe was held in place for 10 min after injection to avoid the blood back streaming. The craniotomy was then sealed with bone wax, and the scalp was closed with sutures. Body temperature was maintained at 37°C throughout the procedure. The mice that died due to anesthesia and unsuccessful ICH models, including asymptomatic and dead mice before euthanasia were excluded from this study. About 5%–10% mice were excluded in study because of the death before sacrifice or without symptoms caused by ICH.

### TAK242 Application

TAK242 (Takeda Pharmaceutical Co) was formulated with 1% dimethyl sulfoxide (σ) and double-distilled water to a final concentration of 0.4 mg/mL and then injected intraperitoneally at a dose of 3 mg/kg beginning 1 h after ICH and giving another injection 6 h after the first injection.

### Tissue Preparation

At the designated time points (6 h, 12 h, 1 day, 3 days, 5 days and 7 days after ICH or sham group and normal brain), the animals were prepared for analysis. To prepare tissues for western blot and real-time PCR (RT-PCR), perihematomal brain specimens were frozen in liquid nitrogen and stored at −80°C until further processing. For immunohistochemistry, the animals were perfused transcardially with 0.9% NS followed by 4% paraformaldehyde in 0.1 M PBS, and the brains were immersed in sucrose and then embedded in paraffin, cut into 6 mm coronal sections, and mounted on coverslips coated with polyL-lysine. For further immunofluorescence staining, the mouse brains were post-fixed for 24 h and then immersed in 0.1 M PBS containing increasing amounts of sucrose (15%–30%) at 4°C for 12 h. The brains were then cut into 30 μm coronal sections.

### RNA Isolation and RT PCR

According to the manufacturer’s instructions, total RNA was isolated from perihematomal brain tissues with a Classic Total RNA isolation kit (Invitrogen, USA). RNA concentrations were determined photometrically by measuring optical density at 260 nm (OD260). Reverse transcript to obtain cDNA using a PrimeScript™ RT Reagent Kit with gDNA Eraser (Takara, Japan). Real-time PCR was conducted in accordance with the instructions of Maxima SYBR Green/ROX qPCR Master Mix (Fermentas, Canada). The experimental data were analyzed using the 2^−ΔΔ^Ct method. All independent experiments were repeated three times. The mRNA expression of genes of interest was normalized by the expression of GAPDH. The primer sequences for mouse NLRP6 inflammasome (forward: 5′AGCTGAGAACGCTGTGTCG3′; reverse: 5′AACTTGGGAACCCCGAAGC3′), and GAPDH (forward: 5′ACCACAGTCCATGCCATCAC 3′; reverse: 5′ACCTTGCCCACAGCCTTG 3′) were synthesized by Invitrogen.

### Western Blot

According to our previous protocol (Wang P. F. et al., 2014; Xiong et al., 2016a), total protein extracts were prepared from perihematomal brain tissues. The proteins were separated on 15% polyacrylamide gels by standard SDS polyacrylamide gel electrophoresis and then transferred to polyvinylidene fluoride (PVDF) membranes (Millipore, USA) using a semidry electroblotting system (Transblot SD, Bio-Rad). The PVDF membranes were blocked with 5% non-fat dry milk in 0.05% Tween-20 in PBS for 1 h at room temperature and were then incubated with a goat polyclonal anti-NLRP6 antibody (1:1000, Santa Cruz, CA, USA), a mouse monoclonal anti-NF-κB p65 (1:1000, Santa Cruz, CA, USA) and a rabbit monoclonal anti-GAPDH antibody (1:1000, Cell Signaling Technology, Danvers, MA, USA) at 4°C overnight. The membranes were further incubated with HRP conjugated goat anti-rabbit secondary antibodies (1:6000, Zhongshan Golden Bridge Inc., China) at 25°C for 1.5 h. Bound antibodies were visualized using a chemiluminescence detection system. The signals were measured by scanning densitometry and computer-assisted image analysis. The optical density of each band was evaluated with ImageJ software. Ratios of NLRP6/GAPDH were calculated from the optical densities.

### Immunohistochemical Staining

According to our previous methods (Fang et al., 2014; Xiong et al., 2016a), the paraffin brain sections were deparaffinized by sequential washing with xylene and graded ethanols. Three-percent hydrogen peroxide was used to block the endogenous peroxidase activity for 10 min, and antigen retrieval was conducted in 10 mmol/l citrate buffer (pH 6.0) for 15 min at 100°C. Then, the sections were treated with 5% bovine serum albumin to block nonspecific staining for 30 min. The sections were then incubated overnight at 4°C with the NLRP6 antibody (1:100, Santa Cruz, CA, USA). Following PBS washing, the sections were incubated with a goat anti-rabbit HRP secondary antibody (1:500, Boster, China) for 1 h at 37°C. All immunostaining was performed at the same time under the same conditions. Three sections were prepared for each brain tissue.

### Immunofluorescent Staining and Analysis

The immunofluorescent staining was performed according to our previously reported methods (Xiong et al., 2016a). Twenty-five-micron brain sections were incubated with the following antibodies: NLRP6 (1:100, Santa Cruz, CA, USA), CD11b (1:200, Santa Cruz, CA, USA), NeuN (1:200, Millipore, USA), GFAP (1:100, Sigma, MO, USA) and CD31 (1:200, Santa Cruz, CA, USA). The secondary Abs included Alexa Fluor 647 (1:200, donkey anti-mouse), Alexa Fluor 594 (1:200, donkey anti-rabbit), and Alexa Fluor 488 (1:200, donkey anti-rabbit, donkey anti-mouse, and donkey anti-rat), all from Invitrogen (Carlsbad, CA, USA). The mature slices were imaged by confocal microscopy (Leica TCS Sp5, Mannheim, Germany). Quantification of double-labeled cells was also performed as previously described (Xiong et al., 2016a), and the results were shown as “Number of cells/per region”. Three sections were prepared for each brain tissue specimen; automatic counting was performed in brain tissues for double-labeled cells, and the averaged data obtained by three researchers who were blinded to the group information represented the value of each brain.

### Enzyme-Linked Immunosorbent Assay (ELISA)

Perihematomal brain tissues collected from each group were homogenized and centrifuged, and the supernatant was collected for analysis. The concentrations of inflammatory factors, including IL-1β, IL-6 and tumor necrosis factor-α (TNF)-α were measured using an enzyme-linked immunosorbent assay (ELISA) reagent kit (Dakewe Biotech Company) following the manufacturer’s instructions.

### Brain Water Content (BMC)

The mice were anesthetized on 1, 3, 5 and 7 days after ICH, and ipsilateral brain tissues were obtained. Then, the brain water content (BMC) was measured according to our previously established methods (Xiong et al., 2016a). Briefly, the obtained brain tissues were dabbed with filter paper to remove surface water and were subsequently evaluated by an electronic balance to determine the wet weight. Then, the brain tissues were dried at 100°C for 24 h to determine the dry weight. The following formula was used to calculate water content: BMC (%) = wet weight − dry weight)/wet weight × 100%.

### Neurological Deficient Score (NDS)

The Neurological Deficient Score (NDS) were measured on 1, 3, 5 and 7 days after ICH according to our previous reports (Wang et al., 2013; Xiong et al., 2016a). Scoring was performed by three trained investigators who were blind to the group information, and the average values were the final score for each mouse.

### Statistical Analysis

All of the data are presented as the mean ± SEM. Analysis was performed using SPSS 13.0 software. The *t*-test for independent samples was used to compare two groups, and comparisons among multiple groups were examined using one-way analysis of variance (ANOVA), followed by Scheffé *post hoc* test. Differences were considered significant at a *P* < 0.05.

## Results

### NLRP6 Inflammasome mRNA and Protein Expression Was Significantly Upregulated in Perihematomal Brain Tissues after ICH

**Figure 1:**
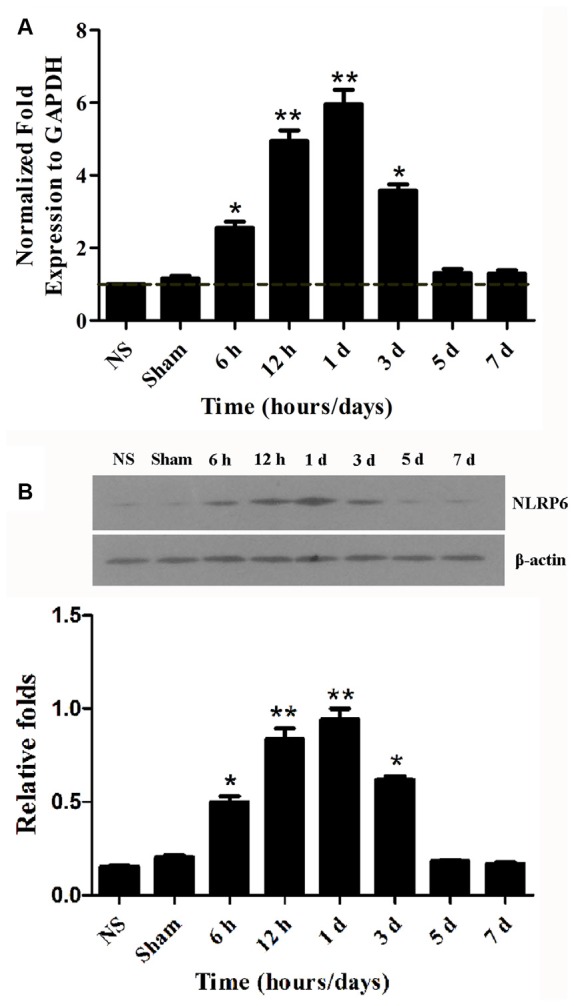
NLRP6 expression in the perihematomal brain tissues after intracerebral hemorrhage (ICH). **(A)** Real-time PCR results showing NLRP mRNA expression in the perihematomal brain tissues of mice at 6 h to 7 days post-ICH. **(B)** Representative bands and densitometric quantification of NLRP6 expression in the perihematomal brain tissues of mice at 6 h to 7 days post-ICH. *n* = 6 per group, **P < 0.01 vs. the normal brain (NS) and Sham groups; *P < 0.05 vs. the NS and Sham groups.

Although NLRP6 inflammasome was first studied almost 10 years ago, little is known about NLRP6 inflammasome in the CNS diseases. To help determine the expression of NLRP6 inflammasome in brain after ICH, we first performed NLRP6 inflammasome mRNA expression profiles of perihematomal brain tissues at different time points. We found that NLRP6 inflammasome expression was markedly increased at 1 day after ICH compared with the normal brain (NS) and sham groups, while the upregulation of NLRP6 inflammasome was gradually decreased from 3 days after ICH (Figure 1A). To confirm the RT-PCR results, we next performed western blot to further measure the NLRP6 inflammasome protein level and found that it was gradually increased in the perihematomal brain tissues at the 6 h, with a peak at 1 day after ICH (Figure 1B).

### Enhanced Expression of NLRP6 Inflammasome Was Mainly Colocalized in GFAP-Positive Astrocytes after ICH

**Figure 2:**
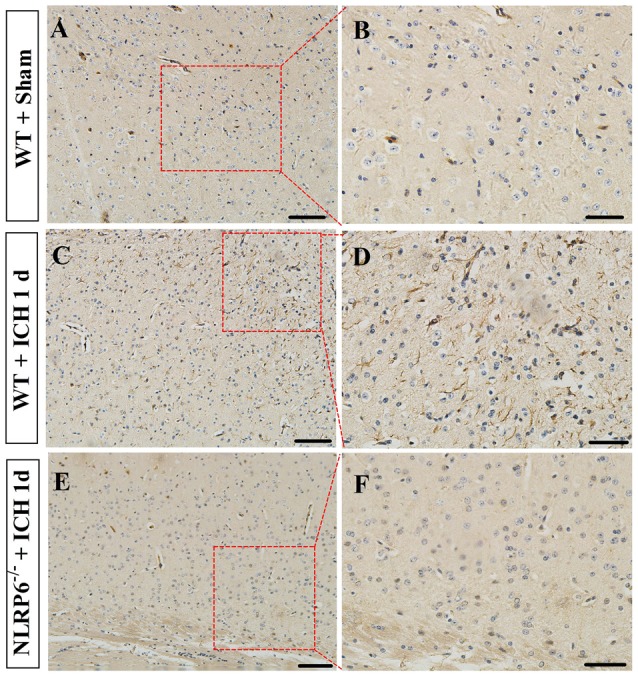
Immunohistochemical staining for NLRP6. **(A,B)** Representative images of NLRP6 immunostaining in the brain tissues in the Sham groups. **(C,D)** Representative images of NLRP6 immunostaining in the perihematomal brain tissues at 1 day after ICH. **(E,F)** Representative images of NLRP6 immunostaining in the brain tissues in the NLRP6^−/−^ mice at 1 day after ICH. *n* = 4 per group, scale bars = 100 μm **(A,C,E)**, scale bars = 50 μm **(B,D,F)**.

**Figure 3:**
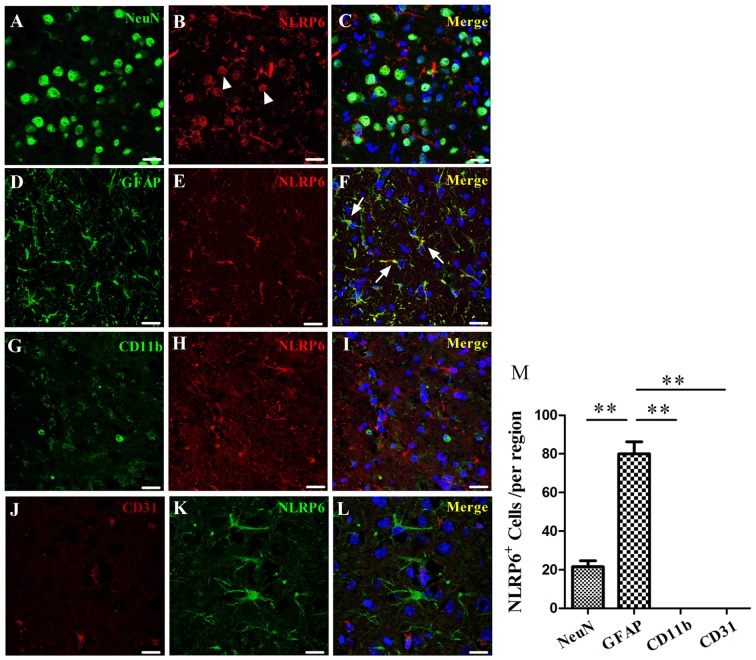
Immunofluorescence staining for NLRP6 after ICH. **(A–C)** Immunofluorescence staining for NeuN **(A)** and NLRP6 **(B)**; the merged image shows that little NLRP6 was expressed in NeuN-positive neurons (**C**; arrow head). **(D–F)** Immunofluorescence staining for glial fibrillary acidic protein (GFAP) **(D)** and NLRP6 **(E)**; the merged image shows that NLRP6 staining was also observed in the cytoplasm and end processes of astrocytes (**F**; arrow).** (G–I)** Immunofluorescence staining for CD11b **(G)** and NLRP6 **(H)**; the merged image shows that NLRP6 was not expressed in CD11b-positive microglia **(I)**. **(J–L)** Immunofluorescence staining for CD31 **(J)** and NLRP6 **(K)**; the merged image shows that NLRP6 was not expressed in CD31-positive endothelial cell **(L)**. All images were captured in the perihematomal brain tissues. **(M)** Densitometric quantification of NLRP6 expression in the perihematomal brain tissues at 1 day after ICH (*n* = 4 per group, **P < 0.01). Scale bars = 25 μm **(A–I)**; = 15 μm **(J–L)**.

Having characterized the expression profiles of NLRP6 inflammasome in the perihematomal brain tissues of mice caused by ICH, we next explored the cell source of NLRP6 inflammasome. First, the results of immunohistochemical staining showed that NLRP6-positive cells were significantly increased in perihematomal brain tissues at 1 day after ICH, and most of the expression occurred in cells with glial-like morphology, while no expression of NLRP6 inflammasome in the NLRP6^−/−^ mice at 1 day after ICH (Figure 2). Immunofluorescence staining was performed to further investigate the cell source of upregulated NLRP6 inflammasome after ICH. To enable direct comparison with the data from the experiment described above, we also performed this experiment at 1 day after ICH. GFAP-positive astrocytes appeared to be the major source of NLRP6 inflammasome expression after ICH, in contrast, little NLRP6 inflammasome expression was detected in NeuN-positive neurons or without expression in CD11b-positive microglia and CD31-positive endothelial cell (Figure 3). While the NLRP6^−/−^ mice showed no NLRP6 inflammasome expression colocalized with GFAP-positive astrocytes after ICH, and little colocalization was detected in the normal brains (Supplementary Figure S1). These results demonstrate that astrocyte-derived NLRP6 inflammasome may be involved in the neuroimmune response to ICH-induced brain injury.

### NLRP6^−/−^ Mice Exhibited Markedly Deterioration of Brain Injury after ICH

**Figure 4:**
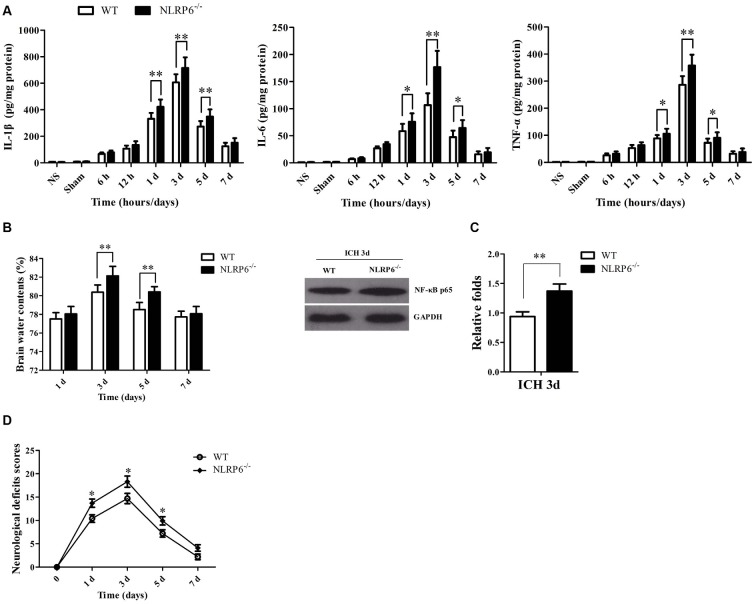
The absence of NLRP6 inflammasome significantly deteriorate the brain injury caused ICH. **(A)** Levels of inflammatory factors in perihematomal brain tissues of mice in the normal brain, sham, 6 h, 12 h, 1 day, 3 days, 5 days and 7 days after ICH (*n* = 6 per group, **P < 0.01; *P < 0.05). **(B)** Brain water content (BMC) in the wild type (WT) and NLRP6^−/−^ mice at 1 day, 3 days, 5 days and 7 days after ICH (*n* = 6 per group, **P < 0.01). **(C)** Representative bands and densitometric quantification of NF-κB p65 expression in the perihematomal brain tissues at 1 day post-ICH between WT and NLRP6^−/−^ mice (*n* = 4 per group, **P < 0.01). **(D)** Neurological Deficient Score (NDS) in the WT and NLRP6^−/−^ mice at 1 day, 3 days, 5 days and 7 days after ICH (*n* = 9 per group, *P < 0.05 vs. the WT group).

Next, we investigated the roles of NLRP6 inflammasome in brain injury caused by ICH. Brain edema, neuroinflammatory levels and NDS are important indexes for assessing ICH-induced brain injury severity. First, we measured the proinflammatory cytokines, including TNF-α, IL-1β and IL-6, at different time points after ICH using the ELISA kits. The results showed that the levels of IL-1β, IL-6 and TNF-α were significantly increased in the NLRP6^−/−^ mice compared with the WT mice at 1, 3 and 5 days after ICH (Figure 4A). Next, we observed BMC in mice after NLRP6 deficiency compared with the WT group at all time points using the dry-wet weight method. We found that BMC in the perihematomal brain tissues on day 3 and 5 were significantly increased in NLRP6^−/−^ mice compared with the WT group after ICH (Figure 4B). Additionally, we found that the NF-κB activity was increased in the NLRP6^−/−^ mice after ICH when compared to the WT mice (Figure 4C). Furthermore, we investigated the biofunctional role of NLRP6 inflammasome in ICH using the NDS. We found that the NDS scores were significantly increased in NLRP6^−/−^ group when compared to the WT group (Figure 4D). These results suggested that enhanced expression of NLRP6 inflammasome protects against the ICH-induced brain injury.

### TLR4 Signaling May Contribute to Increased Expression of NLRP6 Inflammasome after ICH

**Figure 5:**
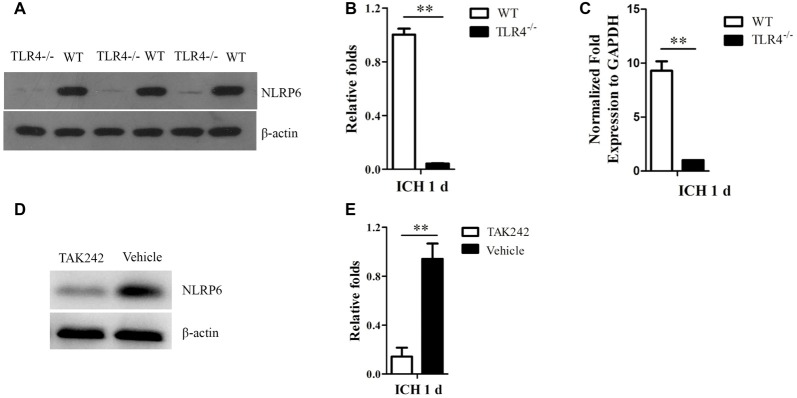
Increased NLRP6 inflammasome expression may be involved in Toll-like receptor 4 (TLR4) signaling after ICH. **(A)** Representative western blot results showing NLRP6 expression between WT and TLR4^−/−^ mice at 1 day after ICH. **(B)** Densitometric quantification of NLRP6 expression in the perihematomal brain tissues of WT and TLR4^−/−^ mice at 1 day after ICH (*n* = 6, **P < 0.01). **(C)** Real-time PCR results showing NLRP6 mRNA expression (*n* = 5, **P < 0.01). **(D)** Representative western blot results showing NLRP6 expression between TAK242 and Vehicle treated mice at 1 day after ICH. **(E)** Densitometric quantification of NLRP6 expression in the perihematomal brain tissues of TAK242 and Vehicle treated mice at 1 day after ICH (*n* = 4, **P < 0.01).

**Figure 6:**
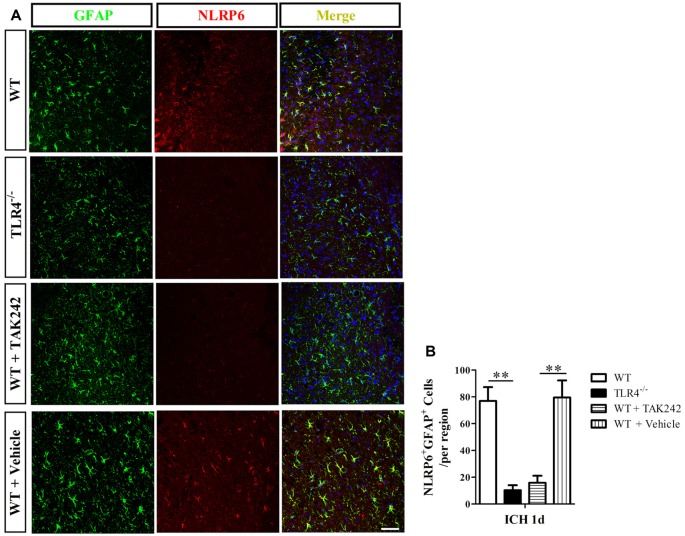
Immunofluorescence staining for NLRP6 inflammasome in TAK242 treated ICH mice. Representative **(A)** and statistical graph **(B)** of immunofluorescent staining of cells expressing NLRP6 and GFAP by astrocytes 1 day after ICH among WT, TLR4^−/−^, WT + TAK242 and WT + Vehicle mice (*n* = 4, **P < 0.01). Scale bars = 50 μm.

Next, we investigated the regulative mechanism of increased NLRP6 inflammasome expression after ICH. As the NLRP6 inflammasome is the intracellular innate immune sensors (Strowig et al., 2012), and the TLR4 is the extracellular innate immune sensors (Akira and Takeda, 2004), which could be triggered by hematoma productions, such as heme, hemoglobin (Hb), etc., after ICH (Lin et al., 2012; Wang Y. C. et al., 2014). Therefore, we want to know whether the extracellular innate immune sensors could influence the intracellular one. Using the TLR4^−/−^ mice, we found that NLRP6 expression was markedly decreased at 1 day after ICH when compared to the WT mice both at protein (Figures 5A,B) and mRNA levels (Figure 5C). The same results were also obtained by using the TLR4 antagonist TAK242 (Figures 5D,E). Furthermore, the results of immunofluorescent staining also showed that double- (NLRP6 and GFAP) positive cells were lower in TLR4^−/−^ and TAK242 treated mice than in C57BL/6 and Vehicle treated mice at 12 h after ICH (Figure 6). These results indicated that enhanced NLRP6 inflammasome expression may via the TLR4 signaling after ICH.

## Discussion

NLRP6 inflammasome is known to be involved in regulating immune responses and promoting periphery nerve recovery. Thus, this study investigated its expression profiles in the perihematomal brain tissues and biofunctional roles after ICH. To our knowledge, we are the first to show that NLRP6 inflammasome expression is significantly increased in perihematomal brain tissues following ICH and that astrocytes are the primary source of NLRP6 inflammasome expression. Moreover, we found that the ICH-induced brain injury was significantly deteriorated when NLRP6 was deficient in mice. These results suggest that increased NLRP6 inflammasome could attenuate ICH-induced brain injury and it may be a novel potential therapeutic target for the treatment of ICH.

Increasing evidence indicates that sterile neuroinflammation is the major reason for the ICH-induced brain injury (Zhou et al., 2014). The extracellular innate immune sensors, such as TLRs, are responsible for receiving the extracellular signaling and activating the glial cells to produce a large number of pro-inflammatory cytokines after ICH (Akira and Takeda, 2004); while the intracellular innate immune sensors, such as NLRP3, NLRP6, etc., are responsible for processing the pro-IL-1β and pro-IL-18 into maturation state to exert important roles in cellular communications (Schroder and Tschopp, 2010; Strowig et al., 2012; Lamkanfi and Dixit, 2014). Previous studies have shown that the microglia-derived NLRP3 inflammasome was significantly increased and contributed to the inflammatory injury by releasing IL-1β and promoting neutrophil infiltration following ICH (Ma et al., 2014), and targeting the NLRP3 inflammasome could reduce the ICH-induced brain injury (Yang et al., 2015; Yuan et al., 2015). While, in our study, we found that the markedly increased NLRP6 inflammasome was mainly colocalized in the GFAP-positive astrocytes, with little expression in neurons while without expression in microglias after ICH, which was inconsistent with the expression profile of NLRP3 inflammasome after ICH (Ma et al., 2014), and we also found that NLRP6 deficiency resulted in the deterioration of the brain injury caused by ICH. These results suggest that in contrast to the NLRP3 inflammasome, the increased NLRP6 inflammasome could reduce the ICH-induced brain injury. Our research results were consistent with the others, for example, the NLRP6 inflammasome has been shown to promote recovery after peripheral nerve injury by dampening inflammatory responses independently of IL-1β and inflammasomes (Ydens et al., 2015); the NLRP6 inflammasome was shown to inhibit colitis and colorectal tumorigenesis (Chen et al., 2011; Elinav et al., 2011; Normand et al., 2011), and dampen inflammatory signaling by negatively regulating canonical NF-κB activation pathway (Anand et al., 2012). These results showed that NLRP6 inflammasome plays a protective role in defending against the damages. However, how the increased NLRP6 inflammasome protected against brain injury caused by ICH needs to be further investigated.

Previously, we have found that the TLR4 signaling was activated after ICH and targeting the TLR4 could attenuate the neuroinflammation related brain injury induced by ICH (Fang et al., 2013; Wang et al., 2013). In addition, Lee et al. (2015) reported that the expression of NLRP6 inflammasome was enhanced when upon LPS stimulation, suggesting that the TLR4 signaling may contribute to the expression of NLRP6 inflammasome, because LPS was considered as one of the most classical damage associated molecular pattern (DAMP) molecules of TLR4 signaling (Akira and Takeda, 2004). In our study, we also found that the increased expression of NLRP6 inflammasome may be attributed to the activated TLR4 signaling after ICH, because the NLRP6 inflammasome was disappeared in TLR4 knockout ICH mice. These results indicated that NLRP6 inflammasome expression may be correlated to the activated TLR4 signaling. While, the exact regulative mechanisms of TLR4 signaling on NLRP6 expression inflammasome after ICH should be further investigated.

In summary, we have provided the first demonstration of the expression and biological roles of NLRP6 inflammasome in perihematomal brain tissue after ICH. The significant upregulation of NLRP6 inflammasome expression in perihematomal brain tissues may be caused by TLR4 signaling protects against ICH-induced brain injury, which suggests that NLRP6 inflammasome may be a novel therapeutic target to reduce the ICH caused disability.

## Author Contributions

P-FW and JC designed the research and wrote the manuscript. Z-GL, YZ, X-HJ, X-WL and A-MZ conducted experiments and analyzed the data.

## Conflict of Interest Statement

The authors declare that the research was conducted in the absence of any commercial or financial relationships that could be construed as a potential conflict of interest.

## Supplementary Material

FIGURE S1 Immunofluorescence staining for NLRP6 inflammasome after intracerebral hemorrhage (ICH) in NLRP6^−/−^ mice and normal brain. Immunofluorescent staining of cells expressing NLRP6 and glial fibrillary acidic protein (GFAP) by astrocytes 1 day after ICH in NLRP6^−/−^ and normal brain (NS; *n* = 4). Scale bars = 50 μm.
