# Supplementary figures and images for: NLRP6 Inflammasome Ameliorates Brain Injury after Intracerebral Hemorrhage
Source: Front Cell Neurosci. 2017 Jul 14;11:206. doi: 10.3389/fncel.2017.00206 (PMC5527702; doi:10.3389/fncel.2017.00206)

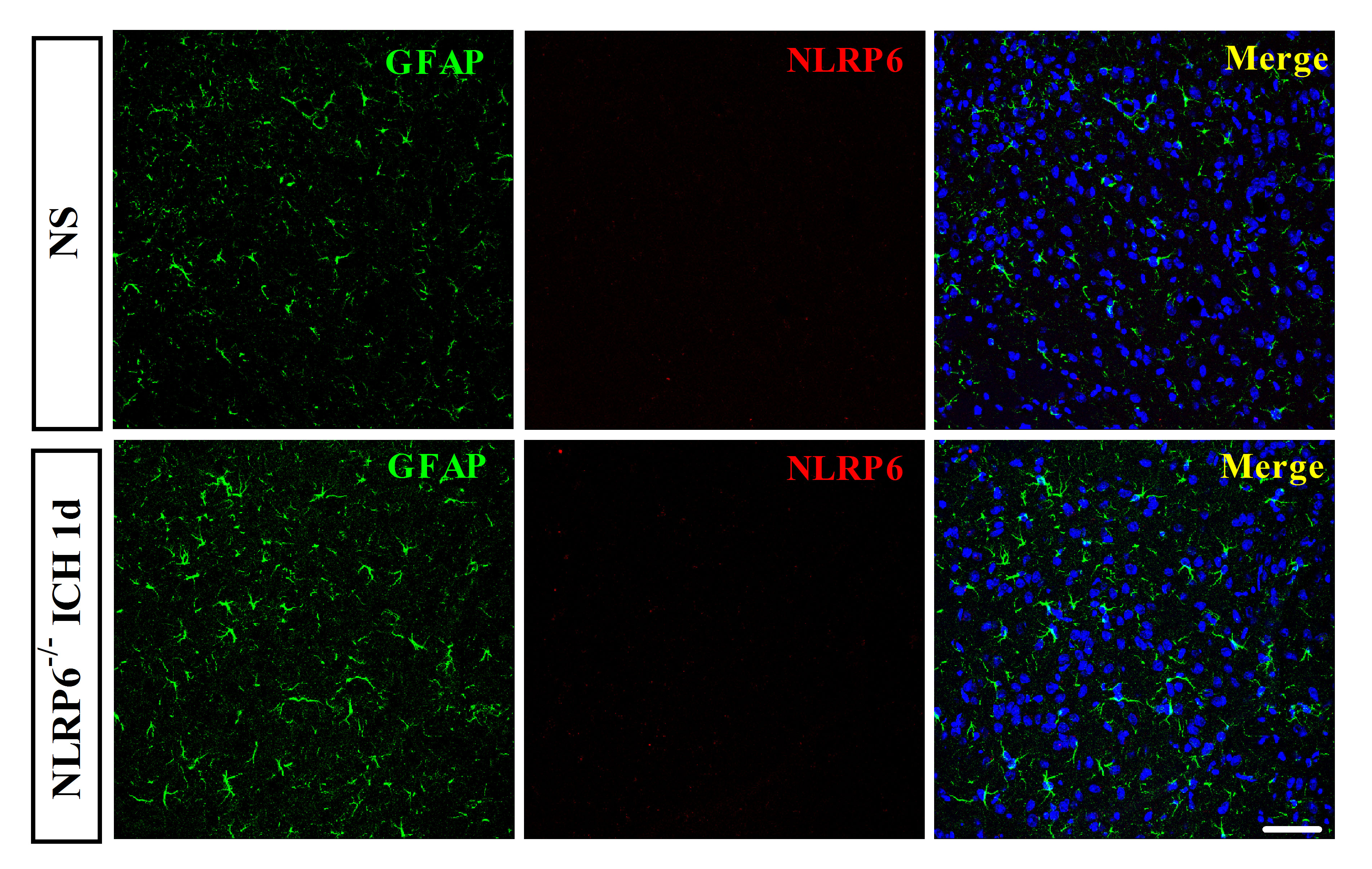

Supplement: FIGURE S1 — Immunofluorescence staining for NLRP6 inflammasome after intracerebral hemorrhage (ICH) in NLRP6−/− mice and normal brain. Immunofluorescent staining of cells expressing NLRP6 and glial fibrillary acidic protein (GFAP) by astrocytes 1 day after ICH in NLRP6−/− and normal brain (NS; n = 4). Scale bars = 50 μm. [file Image_1.jpg]
